# Nutrition for the Prevention and Control of Chronic Degenerative Diseases

**DOI:** 10.3390/nu18213572

**Published:** 2026-10-29

**Authors:** Laura Di Renzo, Paola Gualtieri

**Affiliations:** Section of Food Science, Clinical Nutrition and Pharmaceutical Science, Department of Biomedicine and Prevention, Tor Vergata University of Rome, Montpellier 1, 00133 Rome, Italy; laura.di.renzo@uniroma2.it

In recent decades, the concept of food security has expanded beyond providing enough calories to include nutritional adequacy and dietary quality [1]. Modern diets increasingly include energy-dense, nutrient-poor ultra-processed foods. These foods are often high in free sugars, saturated fats, and refined carbohydrates, while providing relatively little fiber and several essential micronutrients [2,3]. This nutritional transition is associated with the global rise in chronic degenerative diseases (CDDs) [4].

CDDs, including cardiovascular diseases, metabolic syndrome, cancer, and age-related functional decline, are a major public health challenge [5,6]. Their development is influenced by genetic susceptibility, environmental exposures, lifestyle factors, and chronic low-grade inflammation [5,6]. These conditions place a substantial burden on individuals and healthcare systems [5]. The health and economic burden of these conditions highlights the need for multidisciplinary approaches to prevent disease progression and preserve overall health. Within these approaches, nutritional strategies can contribute to the prevention and management of CDDs [7,8].

Nutritional science has expanded beyond the prevention of nutrient deficiencies to play an important role in preventive and precision medicine [7,9,10]. Dietary patterns, bioactive compounds, and targeted nutritional interventions can influence metabolic regulation, gut microbiota composition and function, epigenetic regulation, and immune function [7,9,11].

This Special Issue includes 13 contributions, comprising 10 original research articles and 3 reviews, examining the role of nutrition in different populations and clinical settings. The collection brings together molecular mechanisms, clinical assessment, psychosocial factors, and public health approaches.

The authors of several contributions examine how body composition and metabolic markers can complement conventional anthropometric assessment. Body weight and body mass index (BMI) alone may not adequately describe alterations in body composition, including those associated with sarcopenic obesity [12]. De Lorenzo and colleagues (Contribution 10) investigated the association between sarcopenic obesity and dual-energy X-ray absorptiometry (DXA)-derived visceral adipose tissue (VAT) in adults [13]. Their findings support further investigation of visceral adiposity in the assessment of sarcopenic obesity.

In other contributions, researchers explore how micronutrients may influence molecular pathways. Zinc contributes to cellular signaling and the regulation of several biological processes [14]. Taslim et al. (Contribution 5) reviewed the role of zinc in microRNA (miRNA) biogenesis and signaling in obesity, highlighting how altered zinc status may affect miRNA expression and metabolic regulation. Furthermore, Kim et al. (Contribution 3) examined supplementation with α-lipoic acid, betaine, or L-carnitine in a mouse model of high-fat diet-induced obesity. They observed changes in gut microbiota composition associated with metabolic and adiposity markers, with more pronounced microbial shifts in the L-carnitine group. Human studies show that different dietary fibers can produce distinct microbial and metabolic responses, highlighting the importance of fiber type and individual variability [15].

The interaction between nutritional status, systemic inflammation, and infectious diseases is another major theme of this Special Issue. Several articles address nutritional status and immune responses in the context of the COVID-19 pandemic. Obesity-related metabolic changes can alter immune function and responses to infection [16]. Nigro and colleagues (Contribution 12) reviewed the links between adipocytokines and inflammatory responses during SARS-CoV-2 infection, discussing how obesity-related adipose tissue dysfunction may contribute to disease severity [17]. Addressing vaccine-related side effects, Gualtieri et al. (Contribution 13) examined the association between nutritional supplement intake and reported side effects among Italian adults, observing fewer side effects among supplement users at the end of the vaccination cycle. The maternal–infant relationship during the pandemic was explored by Hochmayr and colleagues (Contribution 9), who measured anti-SARS-CoV-2 antibody titers in breast milk across lactation stages and examined factors associated with their levels. Other researchers have also detected SARS-CoV-2-specific antibodies in human milk after vaccination, with differences according to vaccine type [18].

Micronutrient status and supplement use were also examined in relation to health during and after the pandemic. Grosman-Dziewiszek et al. (Contribution 8) investigated dietary supplement use in Poland, reporting widespread consumption of vitamin D and magnesium and differences according to demographic characteristics. However, widespread supplement use does not establish preventive efficacy. In the CORONAVIT trial, a vitamin D testing and supplementation strategy did not reduce the risk of acute respiratory infections or COVID-19 [19]. Long COVID involves several biological mechanisms and a wide range of persistent symptoms [20]. In a clinical setting, Matsuda and colleagues (Contribution 6) found a high prevalence of vitamin D deficiency among Long COVID outpatients infected during the Omicron phase. Vitamin D deficiency was associated with higher scores for fatigue and depressive symptoms. Pandemic-related changes also raised concerns about pediatric metabolic health: Yuen and colleagues (Contribution 7) reported increased rates of overweight and obesity among children on the autism spectrum during the pandemic, highlighting the need for preventive measures tailored to vulnerable groups. Increases in weight and obesity prevalence during the pandemic have also been reported in broader pediatric populations [21].

Beyond biological mechanisms and immune responses, nutritional choices are also shaped by psychosocial factors [22]. Godos et al. (Contribution 4) examined loneliness, aloneness, and adherence to the Mediterranean diet among older adults in Southern Italy. Their findings linked loneliness and aloneness to a lower likelihood of high adherence, highlighting the importance of considering social connectedness in nutritional interventions [23]. In a related behavioral study, Rodrigues and colleagues (Contribution 2) applied the Theory of Control by Justifications and Immediate Consequences (TJC) to examine inflexibility and disturbed eating attitudes among vegetarian and non-vegetarian university students. Their results suggested an association between inflexibility to change and disturbed eating attitudes. Psychological flexibility has also been studied in relation to disordered eating, although evidence that it acts as a mechanism of change in interventions remains limited [24].

Two contributions focus on nutritional assessment and care in older adults and patients with pancreatic cancer. In older adults, nutritional care should include routine screening and individualized interventions within a multidisciplinary approach [25]. In long-term care settings, Moreno-Guillamont et al. (Contribution 1) developed the VIVA-NIPI score, a weighted multidomain index intended to prioritize nutritional review among institutionalized older adults with overlapping functional, cognitive, nutritional, anthropometric, and biochemical abnormalities. The researchers assessed internal concordance and ranking stability; further independent and external validation is needed before clinical implementation [26]. In oncology, Gualtieri and colleagues (Contribution 11) reviewed the links between diet, gut microbiota, chronic inflammation, and pancreatic ductal adenocarcinoma (PDAC), discussing the potential role of nutritional approaches alongside standard oncological care. Other researchers have also identified fecal microbial signatures associated with PDAC, supporting further investigation of the microbiome as a potential diagnostic resource [27].

In conclusion, the contributions to this Special Issue highlight the value of an integrated, multidimensional approach to the prevention and management of chronic degenerative diseases. Future research should connect molecular findings, including miRNA regulation and gut microbiota modulation, with validated nutritional assessment tools, tailored dietary approaches, and public health strategies. We sincerely thank all the authors, peer reviewers, and the *Nutrients* editorial team for their contributions to this Special Issue.
